# Addressing the Caregiving Crisis

**Published:** 2007-12-15

**Authors:** Rosalynn Carter

**Affiliations:** The Carter Center Mental Health Program

This issue of *Preventing Chronic Disease* (*PCD*) focuses on a set of concerns that is likely to challenge the public's creative spirit and resourcefulness for the next 30 years. Public health is the science and art of preventing disease, prolonging life, and promoting physical and mental health. What we are beginning to see is that success in any one of these areas raises new challenges and presents new problems for us to solve in the other areas. For example, advances in science, better nutrition, and improvements in health care have allowed people around the world to live to unprecedented ages. But this blessing of long life presents us with a new set of formidable challenges: soaring rates of dementia and untreated mental health problems among the elderly, a growing burden of chronic illnesses that affects our communities, disturbing problems of elder abuse, and an unparalleled demand for the services of both professional and family caregivers. All progress comes with costs and challenges, but in the 21st century we will experience this burden on a scale and at a speed that we have never seen before. So, we must prepare ourselves.

I am particularly interested in two issues in this unfolding scenario: mental health and caregiving. When addressing chronic diseases, we must not forget the importance of depression, particularly late-life depression. Depression frequently accompanies chronic illnesses, sometimes emerging as a result of them and other times acting as a risk factor for other illnesses. In either case, depression substantially and independently increases the risk of mortality (1). *PCD* helped address the issue of mental health in its article on The Carter Center Mental Health Program (2); in this editorial, I would like to provide some comments and reflections on the issue of caregiving.

My interest in caregiving goes back to my childhood. I was deeply influenced by how chronic illness affected and shaped my family and by the heroic and selfless efforts of health care providers, including Jimmy's mother, Lillian Carter. She was among the most dedicated and skilled nurses imaginable, and I was in awe of her as I observed the expert care she provided. The type of assistance that Lillian provided as a nurse is increasingly being provided today by family members. In fact, the backbone of our country's long-term, home-based, and community-based care systems is the family caregiver. The approximately 15 million caregivers in the United States provide $306 billion worth of unpaid services each year (3). That amount is almost twice as much as is spent on homecare and nursing home services combined ($158 billion) (4). The number of family caregivers is likely to increase in the upcoming years, as is the intensity of these caregivers' work, not only because of our country's aging population but also because of the changing fabric of our family networks. With the aging baby boomer population, the life expectancy and quality of life in the United States cannot continue to rise, or even remain stable, without increasing the burden on caregivers. But the strains on our society and on these individuals as a result of providing care are becoming apparent:

- A 25-year body of research shows that family caregivers are at risk for a wide range of problems in health and mental health, finances, employment, and retirement. For instance, a recent study found that one-third of family caregivers of people with dementia were depressed (5).
- Caregivers experiencing strain have a 63% higher risk of mortality than noncaregivers, even when adjusting for chronic disease and other risk factors (6).
- Family caregivers are largely neglected by the health and long-term care systems. They frequently are not trained on how to deliver complicated care, not treated as partners in the patient's care, or not encouraged to maintain their own health.
- Professional caregivers work under difficult conditions and are vulnerable to many of the same problems as family caregivers.
- The cost to U.S. businesses attributable to the lost productivity of working caregivers is estimated at between $17.1 billion and $33.6 billion per year and growing (7).

To address this "caregiving crisis," all sectors of society must come together to develop solutions. A broad and coordinated response should address workforce development, community planning, and caregiver education and support, including regulatory and financing issues, more effective use of technology, and development and dissemination of evidence-based practices in caregiving. Building an infrastructure of supports for caregivers will improve caregiver effectiveness and reduce the harm, injury, and burden that can be associated with caregiving in isolation. Most importantly, I believe there must be a fundamental shift in how we value and support caregivers.

I have had a unique opportunity to address the caregiving crisis. With the assistance of many partners, the Rosalynn Carter Institute for Caregiving at Georgia Southwestern State University in Americus, Georgia, was created. Our hope is to play a key role in developing better supports for both family and professional caregivers. As part of our work, we have developed a network of community coalitions (CARE-NETS) that provides a forum for addressing the needs of caregivers in a concerted and coordinated way. In 2007, we launched a new venture. With the support of Johnson & Johnson, the National Quality Care Network (NQCN) was formed to serve as a vehicle for innovation, dissemination, and networking, and to stimulate partnerships for action in our communities. The aim of the NQCN is to support a network of stakeholders in the United States committed to promoting quality in long-term, home-based, and community-based care. Working together with scientists and leaders from many fields, I am very optimistic about our prospects for building communities of care to address the challenges that come with the gift of an aging society.

Visit www.RosalynnCarter.org for more information about the Rosalynn Carter Institute.

## References

[1] Penninx BW, Geerlings SW, Deeg DJ, van Eijk JT, van Tilburg w, Beekman AT (1999). Minor and major depression and the risk of death in older persons. Arch Gen Psychiatry.

[2] Palpant RG, Steimnitz R, Bornemann TH, Hawkins K (2006). The Carter Center Mental Health Program: addressing the public health crisis in the field of mental health through policy change and stigma reduction. Prev Chronic Dis.

[3] Schulz R, Quittner AL (1998). Caregiving for children and adults with chronic conditions: introduction to the special issue. Health Psychol.

[4] Arno PS Economic value of informal caregiving. Proceeding of the Care Coordination and the Caregiving Forum, National Institutes of Health, Department of Veterans Affairs.

[5] Covinsky KE, Newcomer R, Fox P, Wood J, Sands L, Dane K (2003). Patient and caregiver characteristics associated with depression in caregivers of patients with dementia. J Gen Intern Med.

[6] Schulz R, Beach SR (1999). Caregiving as a risk factor for mortality: the Caregiver Health Effects Study. JAMA.

[7] (2006). The MetLife caregiving cost study: productivity losses to U.S. business.

